# Renal Resistive Index and Cardiovascular Events, Cardiovascular Mortality, and All-Cause Mortality: Protocol for a Systematic Review and Meta-Analysis

**DOI:** 10.2196/79071

**Published:** 2025-12-31

**Authors:** Giulio Geraci, Vincenzo Calabrese, Pietro Ferrara, Giusy Rita Maria La Rosa, Giuseppe Cuttone, Luigi La Via, Nicola Sinatra, Alessandra Sorce, Caterina Carollo, Giuseppe Mulè, Jacob George, Riccardo Polosa

**Affiliations:** 1 Department of Medicine and Surgery, University of Enna Kore, Enna, Italy; 2 Center for Public Health Research, University of Milan–Bicocca, Monza, Italy; 3 Laboratory of Public Health, IRCCS Istituto Auxologico Italiano, Milan, Italy; 4 Department of Clinical and Experimental Medicine, University of Catania, Via Santa Sofia 78, Catania, 95123, Italy, 39 095 4781124; 5 Department of Anesthesia and Intensive Care 1, University Hospital Policlinico “G Rodolico-San Marco”, Catania, Italy; 6 Nephrology and Dialysis Unit, Paolo Borsellino Hospital, Marsala, Italy; 7 Department of Health Promotion, Mother and Child Care, Internal Medicine and Medical Specialties, Unit of Nephrology and Dialysis, University of Palermo, Palermo, Italy; 8 European Society of Hypertension Excellence Center, Chair of Nephrology, University of Palermo, Palermo, Italy; 9 University of Dundee Medical School, Ninewells Hospital, Dundee, United Kingdom; 10 Centre of Excellence for the Acceleration of HArm Reduction (CoEHAR), University of Catania, Catania, Italy

**Keywords:** all-cause mortality, cardiovascular outcomes, meta-analysis, renal resistive index, systematic review, PRISMA

## Abstract

**Background:**

The renal resistive index (RRI) is a noninvasive indicator of renal vascular resistance and systemic hemodynamic status. Elevated RRI values have been consistently associated with subclinical vascular damage and target organ injury. Observational studies within the past decade have suggested that RRI may also serve as a prognostic marker for adverse cardiovascular outcomes and mortality. However, the evidence remains scattered and heterogeneous, and no systematic review has yet synthesized this body of literature.

**Objective:**

This systematic review aims to evaluate the association between elevated RRI and the risk of cardiovascular events, cardiovascular mortality, and all-cause mortality in adult populations.

**Methods:**

This protocol has been developed in accordance with the PRISMA-P (Preferred Reporting Items for Systematic Reviews and Meta-Analyses Protocols) guidelines. We will include observational studies (prospective and retrospective cohort studies and nested case-control studies) involving adults (≥18 years) with RRI measurements obtained through Doppler ultrasound. Studies focused on pediatric populations, pregnant women, and patients undergoing dialysis will be excluded. The primary exposure will be elevated RRI, typically defined as RRI of ≥0.70, compared to lower or normal values. The primary outcomes are cardiovascular events, cardiovascular mortality, and all-cause mortality with a minimum follow-up of 6 months. A comprehensive search will be conducted in PubMed, Embase, Web of Science, and Scopus, as well as in gray literature sources. Two independent reviewers will screen articles, extract data, and assess risk of bias using Version 2 of the Cochrane revised risk-of-bias tool for randomized controlled trials and the Risk of Bias in Nonrandomized Studies of Interventions and Risk of Bias in Nonrandomized Studies of Exposure tools for nonrandomized studies of interventions and exposure, respectively. Meta-analyses will be conducted if at least 3 studies report comparable data, and effect estimates will be calculated using raw data whenever possible. Subgroup and meta-regression analyses will be used to explore heterogeneity, whereas sensitivity analyses will be conducted to assess the robustness of the observed results. The Grading of Recommendations Assessment, Development, and Evaluation framework will be applied to evaluate the overall quality of evidence.

**Results:**

A preliminary exploratory search has been conducted to map the existing literature and confirm the absence of prior systematic reviews on this topic. The formal study selection and data extraction are expected to begin upon protocol acceptance with review completion anticipated by December 2026.

**Conclusions:**

By systematically synthesizing the available literature, this review will provide a comprehensive overview of the prognostic value of RRI in predicting cardiovascular outcomes and mortality. The findings may inform clinical decision-making, enhance cardiovascular risk stratification, and identify research gaps for future studies focused on standardizing RRI assessment and its clinical applications.

## Introduction

### Background

The renal resistive index (RRI) is a noninvasive Doppler-derived hemodynamic parameter first introduced in the 1980s to evaluate intrarenal vascular resistance. Since then, its applications have progressively expanded from nephrology to cardiology and critical care. RRI is calculated as (peak systolic velocity – end-diastolic velocity)/peak systolic velocity and reflects both renal vascular resistance and vascular compliance. This measurement captures the dynamics of renal blood flow and is influenced by intrarenal and systemic factors, including arterial stiffness, vascular tone, and parenchymal compliance. In healthy individuals, typical RRI values range from 0.60 to 0.70, whereas higher values indicate increased vascular stiffness and reduced renal compliance [1]. Over the past 2 decades, RRI has emerged as a potential marker of subclinical vascular dysfunction and organ damage, particularly in patients with arterial hypertension, chronic kidney disease, and diabetes [2-4].

Elevated RRI values—typically defined as ≥0.70—have been consistently associated with early signs of end-organ damage, such as carotid intima-media thickness, carotid atherosclerosis, aortic stiffness, left ventricular hypertrophy, and albuminuria. These markers are well-established predictors of adverse cardiovascular outcomes and mortality in diverse clinical settings [4-11]. More recently, the literature has highlighted that RRI reflects not only renal vascular resistance but also systemic cardiovascular properties such as arterial stiffness and pulse pressure. In this regard, higher RRI values have been linked to increased cardiovascular morbidity and mortality in older adults followed for 7 years [12]. Similarly, in patients with primary hypertension and no prior cardiovascular disease, RRI emerged as an independent predictor of both renal and cardiovascular events over a mean follow-up of 3.1 years, particularly when combined with reduced glomerular filtration rate [4,13].

These findings collectively highlight the potential utility of RRI as a prognostic tool in cardiovascular risk stratification. However, the existing literature remains fragmented, with variations in study populations, RRI thresholds, outcome definitions, and follow-up durations. As a result, the strength and consistency of the association between elevated RRI and major clinical outcomes—including cardiovascular events, cardiovascular mortality, and all-cause mortality—remain unclear.

To date, no systematic review has comprehensively evaluated this association across different populations and clinical settings. Therefore, a structured synthesis of the available evidence is warranted to clarify the prognostic value of RRI and inform its potential application in clinical practice.

### Study Objectives

The aim of this systematic review is to evaluate the impact of elevated RRI on the risk of cardiovascular events, cardiovascular mortality, and all-cause mortality in adult populations. By synthesizing current evidence, this review will clarify the prognostic utility of RRI and help inform its potential role in clinical risk stratification.

## Methods

This systematic review protocol follows the PRISMA-P (Preferred Reporting Items for Systematic Reviews and Meta-Analyses Protocols) guidelines [14]. The protocol for this review has been registered in the PROSPERO database under the number CRD420251071996.

### Eligibility Criteria

#### Type of Participants

This review will include studies involving adult participants (aged ≥18 years) in whom the RRI was measured using Doppler ultrasound. Participants may come from various clinical settings, including the general population and outpatient clinics, and may have conditions such as hypertension, kidney disease, or heart failure (HF). We will exclude studies involving children or adolescents, as well as those focused on pregnant women due to physiological changes during pregnancy that can affect pathophysiological pathways and RRI values. Importantly, eligible participants must have undergone RRI assessment in nonhospitalized, stable conditions. Patients who experienced prior hospitalizations (eg, for HF decompensation, myocardial infarction, or other acute events) will still be eligible provided that the baseline RRI measurement was performed in an ambulatory setting and at least 3 months after the last hospitalization. This criterion is intended to ensure that the RRI reflects a patient’s chronic hemodynamic status rather than transient alterations related to acute illness or hospitalization. In this way, the review will specifically assess the role of RRI in long-term cardiovascular risk stratification rather than in the acute setting. Studies exclusively including acute unstable hospital inpatients, as well as patients receiving maintenance dialysis (hemodialysis or peritoneal dialysis), will be excluded as RRI in these settings is influenced by specific hemodynamic factors that differ from those in nonhospitalized or nondialysis populations; this may introduce confounding, limit clinical interpretability, and inflate effect sizes due to their high-risk status [4].

#### Type of Exposure and Comparators

The exposure of interest in this review is an elevated RRI as measured via Doppler ultrasound. RRI will be analyzed both as a continuous measure and by categorical thresholds. In the latter case, we will consider studies that define elevated RRI using a threshold value—commonly RRI of ≥0.70—or other cutoff points as specified in the individual studies. Participants with elevated RRI will be compared to those with normal or lower RRI values, typically defined as RRI of <0.70. We will include all studies that clearly categorize participants into groups based on higher versus lower RRI values, regardless of the exact threshold used, if the definitions are clearly reported.

#### Type of Outcomes

The primary outcomes of interest in this review are cardiovascular events, cardiovascular mortality, and all-cause mortality. In particular, cardiovascular outcomes will be prespecified into the following categories: (1) major adverse cardiovascular events (myocardial infarction, stroke, and cardiovascular death), (2) HF outcomes (eg, hospitalization for HF), and (3) other cardiovascular outcomes (eg, arrhythmias, revascularization, or composite outcomes as reported). When studies use heterogeneous or composite definitions, outcomes will be harmonized into these categories, and sensitivity analyses will assess the impact of different definitions on pooled estimates.

Cardiovascular mortality refers to death specifically caused by cardiovascular diseases, whereas all-cause mortality includes death from any cause. If available, we will also consider secondary outcomes such as individual cardiovascular end points or composite outcomes that combine multiple clinical events. We will include studies that report at least one of these outcomes in relation to RRI values and that have a minimum follow-up period of 6 months to ensure sufficient time for outcome development and capture meaningful prognostic associations.

#### Type of Studies

This review will include observational studies, such as prospective cohort studies, retrospective cohort studies, and nested case-control studies, that examine the association between RRI and the outcomes of interest. Randomized controlled trials (RCTs) reporting relevant data on RRI will also be included. We will exclude case reports, case series, letters, editorials, conference abstracts, and review articles as these do not provide primary data suitable for quantitative or qualitative synthesis.

### Search Strategy

Published studies will be identified through a comprehensive search of major international databases: PubMed, Embase, Web of Science, and Scopus. A detailed search strategy for each database will be reported in the appendix or supplementary materials (Multimedia Appendix 1). To ensure a comprehensive capture of relevant evidence, we will complement database searches with a structured gray literature search, including OpenGrey, ClinicalTrials.gov, and the World Health Organization International Clinical Trials Registry Platform. The reference lists of the included studies and relevant reviews will also be manually screened. The search will cover all literature available up to the date of the final search, with no restrictions on publication language, publication year, or geographic location.

The literature search will be performed using a combination of MeSH (Medical Subject Headings; in PubMed only), keywords, and free-text terms related to “renal resistive index,” “RRI,” “renal Doppler,” “cardiovascular events,” “mortality,” and “outcomes.” The search strategy will be tailored to the specific syntax and indexing of each database to ensure comprehensive retrieval of relevant studies.

### Study Selection

All records identified through the database searches will be imported into a reference management software, where duplicate entries will be automatically removed. The study selection process will be carried out independently by 2 reviewers in 2 stages. First, titles and abstracts will be screened to identify potentially relevant studies. Full-text articles of selected records will then be retrieved and assessed for eligibility based on the predefined inclusion criteria. Any disagreements between reviewers will be resolved through discussion or consultation with a third member of the review team. Studies deemed eligible after full-text screening will be included in the systematic review and, where appropriate, in the meta-analysis. The overall selection process, along with reasons for exclusion at the full-text stage, will be documented and presented in a PRISMA (Preferred Reporting Items for Systematic Reviews and Meta-Analyses) flow diagram [14].

### Data Extraction

Data will be extracted independently by 2 reviewers using a standardized and piloted extraction form. To ensure accuracy and completeness, all extracted data will be cross-verified against the original full-text articles by a third reviewer. Any disagreements or uncertainties will be resolved through discussion among the study team. If key information is missing or unclear, the corresponding authors of the included studies will be contacted via email, with up to 3 attempts made over a 3-month period, to obtain the necessary data.

The data extracted will be directly aligned with the research questions of this review. Specifically, the following information will be collected from each included study: general information (ie, first author, year of publication, and country of study), study characteristics (study design, study period, and duration of follow-up), participant characteristics (sample size, mean or median age, sex distribution, and relevant comorbidities), exposure details (RRI values, cutoff points used to define elevated RRI, and methodology for RRI assessment), and outcomes (cardiovascular events and mortality, including definitions and tools or criteria used to assess outcomes).

### Risk-of-Bias Assessment

The risk of bias for each included study will be assessed independently by 2 reviewers. For RCTs, we will use version 2 of the Cochrane risk-of-bias tool for randomized trials. For nonrandomized studies, we will apply the Risk of Bias in Nonrandomized Studies of Interventions (ROBINS-I) and Risk of Bias in Nonrandomized Studies of Exposure (ROBINS-E) tools to assess risk of bias in studies of interventions and exposure, respectively [15]. Any disagreements between the reviewers will be resolved through discussion and, if necessary, consultation with a third reviewer. To facilitate clear presentation of the findings, we will use the *robvis* tool to generate graphical summaries of the risk-of-bias assessments [16].

### Data Synthesis and Analysis

Data synthesis will be conducted using statistical software commonly used for systematic reviews and meta-analyses, including Review Manager (version 5.4.1; The Cochrane Collaboration) and the R software (version 4.2.3; R Foundation for Statistical Computing).

Meta-analyses will be performed for each outcome if at least 3 studies provide sufficient and comparable data. Pooled effect estimates (and 95% CIs) will be calculated using either a fixed-effects or random-effects model depending on the degree of heterogeneity across studies. The primary analysis will assess the association between RRI and the outcomes of interest. Whenever possible, we will analyze RRI as a continuous variable to minimize the impact of arbitrary cutoffs. In addition, categorical analyses will be performed using study-defined thresholds, with results presented according to commonly used definitions (eg, ≥0.70). The primary effect measures will be risk ratios, hazard ratios, or odds ratios depending on the type of study design. When possible, effect estimates will be calculated directly from the raw data in line with Cochrane Handbook for Systematic Reviews of Interventions guidance [15]. If raw data are not available, separate meta-analyses will be conducted by effect measure, and narrative synthesis will be used to summarize findings. Separate meta-analyses will be also conducted for observational studies and RCTs to ensure methodological consistency [17,18]. Subgroup analyses will be conducted only when at least 3 studies provide data for the subgroup of interest to ensure sufficient robustness and interpretability. In particular, we will conduct subgroup analyses stratified by cutoff values to explore whether different thresholds explain the heterogeneity in effect estimates. Other subgroups may include variables such as age, sex, presence of arterial hypertension, and different RRI thresholds. Sensitivity analyses will be conducted using a leave-one-out approach in which each study is sequentially removed to assess the influence of individual studies on the overall effect estimate. Differences between the recalculated and original pooled estimates will be compared to evaluate the robustness of the results.

Heterogeneity will be assessed using the chi-square test and the *I*^2^ statistic. A *P* value of <.10 in the chi-square test or an *I*^2^ value of >50% will be considered indicative of significant heterogeneity. Conversely, a *P* value of ≥.1 and *I*^2^ of ≤50% will be interpreted as evidence of low or no significant heterogeneity. If heterogeneity exceeds 75%, potential sources will be explored using subgroup analyses and meta-regression. In particular, if 10 or more studies are available for a given outcome, meta-regression analyses will be performed to further investigate sources of heterogeneity and determine whether specific study-level variables explain variations in effect estimates—to either reconcile conflicting findings or reinforce consistent associations [18]. Moderating variables (ie, the extracted studies’ characteristics) will be initially assessed separately in univariable models before being examined together in a single model.

For outcomes for which meta-analysis is not feasible, a narrative synthesis will be provided, summarizing study characteristics, populations, exposure definitions, and key findings.

### Assessment of the Certainty of the Evidence

The overall quality of evidence for each primary outcome will be assessed using the Grading of Recommendations Assessment, Development, and Evaluation (GRADE) approach. The certainty of the evidence will be rated as high, moderate, low, or very low based on 5 key domains: study design, risk of bias, inconsistency, indirectness, and imprecision. In applying the GRADE approach, we anticipate that the domains of risk of bias and indirectness will be most critical in nonrandomized studies of RRI. Risk of bias will be evaluated using the ROBINS-I and ROBINS-E tools, as mentioned previously. Indirectness will be assessed in relation to variability in RRI thresholds, study populations, and outcome definitions. These factors will be explicitly reported in a summary of findings table to clearly present the quality of evidence and the key results for each outcome included in the review [19].

## Results

As of June 2025, a preliminary scoping search has been conducted to assess the type and volume of available evidence and ensure that no prior systematic reviews or meta-analyses have addressed this specific research question. The formal literature search will begin upon acceptance of this protocol, with study selection and data extraction expected to be completed within 6 months and data synthesis and final manuscript preparation expected to be completed by December 2026, ensuring the timely completion of the review. Clinicians (nephrologists and cardiologists) will contribute to the interpretation of findings, whereas input from patient advocacy groups will be sought to guide dissemination strategies and ensure that results are aligned with patient needs and perspectives.

## Discussion

### Expected Principal Findings

This systematic review is expected to demonstrate that elevated RRI is associated with an increased risk of cardiovascular events, cardiovascular mortality, and all-cause mortality across diverse adult populations. By considering both continuous and categorical definitions of RRI, the review will provide insights into the consistency and magnitude of this association and clarify whether different thresholds meaningfully influence prognostic value. Furthermore, the planned subgroup, sensitivity, and meta-regression analyses will allow us to explore whether population characteristics (eg, hypertension, chronic kidney disease, and age) or methodological heterogeneity explain differences across studies.

### Comparison to Prior Work

Although multiple observational studies have reported an association between elevated RRI and adverse cardiovascular outcomes [4,20,21], the evidence base remains fragmented and heterogeneous. Previous studies have used varying thresholds for RRI and have reported on diverse cardiovascular end points, often without harmonization across outcomes [22-24]. To date, no systematic review or meta-analysis has comprehensively synthesized these findings. By consolidating the available evidence, our review will build on individual studies and provide the first quantitative estimate of the prognostic value of RRI in predicting cardiovascular and mortality outcomes. In addition, our approach aligns with recent systematic review protocols in the field while offering unique contributions through broader inclusion criteria, structured outcome classification, and sensitivity analyses addressing high-risk groups. Importantly, this systematic review does not overlap with other recently registered or published protocols in similar domains and will provide a distinct and nonduplicative contribution to the literature.

### Strengths and Limitations

A major strength of this review is the comprehensive search strategy, which includes multiple databases, structured gray literature sources, and preprint servers, thereby reducing the risk of publication bias. The use of version 2 of the Cochrane risk-of-bias tool for randomized trials and the ROBINS-I and ROBINS-E tools for nonrandomized studies, together with the GRADE framework, will ensure rigorous evaluation of evidence quality. The inclusion of subgroup and meta-regression analyses will enhance the interpretability of the findings.

Nevertheless, certain limitations are anticipated. Variability in RRI thresholds and outcome definitions may also introduce heterogeneity; however, these issues will be explicitly addressed through prespecified outcome groupings and methodological strategies. Finally, the observational nature of most eligible studies may result in residual confounding that cannot be fully eliminated.

### Future Directions

If elevated RRI is confirmed to be a robust predictor of cardiovascular and mortality outcomes, this parameter could be incorporated into cardiovascular risk stratification models and support clinical decision-making for high-risk patients [25]. Future research should prioritize the standardization of RRI measurement protocols; the definition of clinically meaningful thresholds; and the validation of its prognostic role in prospective cohorts across diverse populations, including patients undergoing dialysis and inpatients who are acutely ill. This direction is consistent with recent recommendations from the critical care field that emphasize the need for standardized RRI assessment to enhance reliability and facilitate clinical translation. Furthermore, cost-effectiveness analyses may be warranted to determine the potential health care impact of integrating RRI into routine practice.

### Dissemination Plan

The results of this systematic review will be disseminated through publication in a peer-reviewed journal and presentations at relevant scientific conferences in nephrology, cardiology, and internal medicine. Summaries of the findings will be shared with professional societies and patient advocacy groups to facilitate translation into clinical practice. In line with JMIR guidance, dissemination will also include digital platforms and stakeholder engagement to maximize accessibility and impact for clinicians, researchers, and patients.

### Conclusions

In summary, this systematic review will provide the first comprehensive synthesis of the evidence regarding the prognostic role of RRI in predicting cardiovascular and mortality outcomes. By systematically addressing methodological heterogeneity and assessing evidence quality, the review aims to clarify the clinical utility of RRI, inform risk prediction strategies, and highlight priorities for future research.

## Supplementary material

10.2196/79071 Multimedia Appendix 1 Search strategy for each database.

## Abbreviations

GRADE: Grading of Recommendations Assessment, Development, and Evaluation
HF: heart failure
MeSH: Medical Subject Headings
PRISMA: Preferred Reporting Items for Systematic Reviews and Meta-Analyses
PRISMA-P: Preferred Reporting Items for Systematic Reviews and Meta-Analyses Protocols
RCT: randomized controlled trial
ROBINS-E: Risk of Bias in Nonrandomized Studies of Exposure
ROBINS-I: Risk of Bias in Nonrandomized Studies of Interventions
RRI: renal resistive index
