# Supplementary material for: Renal Resistive Index and Cardiovascular Events, Cardiovascular Mortality, and All-Cause Mortality: Protocol for a Systematic Review and Meta-Analysis
Source: JMIR Res Protoc. 2025 Dec 31;14:e79071. doi: 10.2196/79071 (PMC12755896; doi:10.2196/79071)
Supplement: Multimedia Appendix 1 [file resprot-v14-e79071-s001.docx]

**Multimedia Appendix 1**. Search strategy for each database

| **PUBMED** |
| --- |
| "renal resistive index"[All Fields] OR (((((((((((((("renal"[All Fields] OR "renals"[All Fields]) AND ("resist"[All Fields] OR "resistance"[All Fields] OR "resistances"[All Fields] OR "resistant"[All Fields] OR "resistants"[All Fields] OR "resisted"[All Fields] OR "resistence"[All Fields] OR "resistences"[All Fields] OR "resistent"[All Fields] OR "resistibility"[All Fields] OR "resisting"[All Fields] OR "resistive"[All Fields] OR "resistively"[All Fields] OR "resistivities"[All Fields] OR "resistivity"[All Fields] OR "resists"[All Fields]) AND ("abstracting and indexing"[MeSH Terms] OR ("abstracting"[All Fields] AND "indexing"[All Fields]) OR "abstracting and indexing"[All Fields] OR "index"[All Fields] OR "indexed"[All Fields] OR "indexes"[All Fields] OR "indexing"[All Fields] OR "indexation"[All Fields] OR "indexations"[All Fields] OR "indexe"[All Fields] OR "indexer"[All Fields] OR "indexers"[All Fields] OR "indexs"[All Fields])) AND "or"[All Fields]) AND (("renal"[All Fields] OR "renals"[All Fields]) AND ("haemodynamic"[All Fields] OR "hemodynamics"[MeSH Terms] OR "hemodynamics"[All Fields] OR "hemodynamic"[All Fields] OR "haemodynamical"[All Fields] OR "haemodynamically"[All Fields] OR "haemodynamics"[All Fields] OR "hemodynamical"[All Fields] OR "hemodynamically"[All Fields]))) AND "or"[All Fields]) AND ("read res instr"[Journal] OR "rri"[All Fields])) AND "and"[All Fields]) AND ("CV"[All Fields] AND ("event"[All Fields] OR "event s"[All Fields] OR "events"[All Fields]))) AND "or"[All Fields]) AND (("cardiovascular system"[MeSH Terms] OR ("cardiovascular"[All Fields] AND "system"[All Fields]) OR "cardiovascular system"[All Fields] OR "cardiovascular"[All Fields] OR "cardiovasculars"[All Fields]) AND ("event"[All Fields] OR "event s"[All Fields] OR "events"[All Fields]))) AND "or"[All Fields]) AND ("mortality"[MeSH Terms] OR "mortality"[All Fields] OR "mortalities"[All Fields] OR "mortality"[MeSH Subheading])) AND "mesh or"[All Fields]) AND (("cardiovascular system"[MeSH Terms] OR ("cardiovascular"[All Fields] AND "system"[All Fields]) OR "cardiovascular system"[All Fields] OR "cardiovascular"[All Fields] OR "cardiovasculars"[All Fields]) AND ("mortality"[MeSH Terms] OR "mortality"[All Fields] OR "mortalities"[All Fields] OR "mortality"[MeSH Subheading]))) |
| **EMBASE** |
| ('renal resistive index'/exp OR 'renal resistive index':ab,ti OR 'renal resistance index':ab,ti OR 'renal hemodynamics':ab,ti OR rri:ab,ti)  AND  ('cardiovascular event'/exp OR 'cardiovascular events':ab,ti OR 'cv event':ab,ti OR 'cardiovascular mortality'/exp OR 'cardiovascular mortality':ab,ti OR 'all-cause mortality':ab,ti OR mortality:ab,ti) |
| **SCOPUS** |
| TITLE-ABS-KEY ( ( ( "renal resistive index" ) OR ( "Renal resistance index" ) OR ( "renal hemodynamics" ) OR ( rri ) ) AND ( ( "cardiovascular events" ) OR ( mortality ) OR ( "all-cause mortality" ) OR ( "Cardiovascular mortality" ) ) ) |
| **WEB OF SCIENCE** |
| ("renal resistive index" OR "renal resistance index" OR "renal hemodynamics" OR RRI) (All Fields) and ("cardiovascular events" OR "CV events" OR "cardiovascular mortality" OR "all-cause mortality" OR mortality) (All Fields) |
